# Harnessing genomics and translational research to improve health in Africa: a report of the 13^th^ African Society of Human Genetics meeting in Dar es Salaam, Tanzania

**DOI:** 10.11604/pamj.2024.49.79.42550

**Published:** 2024-11-14

**Authors:** Siana Nkya, Aneth David, Mohamed Zahir Alimohamed, Kilaza Samson, Grantina Modern, Michèle Ramsay, Julie Makani, Scott Williams, Victoria Nembaware, Ambroise Wonkam

**Affiliations:** 1 Tanzania Human Genetics Organisation (THGO), Dar es Salaam, Tanzania,; 2 Department of Haematology and Blood Transfusion, Muhimbili University of Health and Allied Sciences, Dar es Salaam, Tanzania,; 3 Sickle Cell Program, Muhimbili University of Health and Allied Sciences, Dar es Salaam, Tanzania,; 4 Department of Biochemistry and Molecular Biology, Muhimbili University of Health and Allied Sciences, Dar es Salaam, Tanzania,; 5 Plant Protection Department, Swedish University of Agricultural Sciences, Alnarp, Sweden,; 6 Department of Molecular Biology and Biotechnology, University of Dar es Salaam, Tanzania Plant, Dar es Salaam, Tanzania,; 7 Department of Genetics, University Medical Centre Groningen, University of Groningen, Groningen, The Netherlands,; 8 Department of Science and Laboratory Technology, Dar es Salaam Institute of Technology, Dar es Salaam, Tanzania,; 9 Nelson Mandela Institute of Technology, Arusha, Tanzania,; 10 Sydney Brenner Institute for Molecular Bioscience, Faculty of Health Sciences, University of the Witwatersrand, Johannesburg, South Africa,; 11 Department of Population and Quantitative Health Sciences and Genetics and Genome Sciences, Cleveland Institute for Computational Biology, Case Western Reserve University, School of Medicine, Cleveland, United States of America,; 12 African Society of Human Genetics, Division of Human Genetics, Department of Pathology, Faculty of Health Sciences, University of Cape Town, 1 Anzio Road, Observatory, 7925, Cape Town, South Africa,; 13 McKusick-Nathans Institute of Genetic Medicine and the Department of Genetic Medicine at Johns Hopkins University School of Medicine, Baltimore, United States of America

**Keywords:** Human genetics, Human Genetics Africa, Human Genetics Tanzania

## Abstract

The thirteenth conference of the African Society of Human Genetics with the theme “harnessing genomics and translational research to improve health in Africa” was held in Dar es Salaam, Tanzania, in August 2021, using a hybrid in-person and virtual model for participation in the wake of COVID-19 pandemic. During the meeting, African research across various human genetics disciplines was presented, including talks on the genetics of infectious and non-communicable diseases, population genetics, and translational research. The meeting also featured presentations on pharmacogenomics, genetics of developmental disorders, cancer genetics and genetics of rare diseases. In-depth discussions on ethical legal and social issues in genomics research and community and patient engagement were also key sessions of this meeting. The primary focus of the conference and the discussions was how to translate the wealth of genomic research in the continent into improved health outcomes in the continent. In this report, we summarize the key scientific research relevant to Africa presented and discussed during the meeting providing an overview of the progress of human genetics in the continent. We also discuss opportunities and challenges of harnessing genomics for health improvement in Africa.

## Conference proceedings

**Figure 1:**
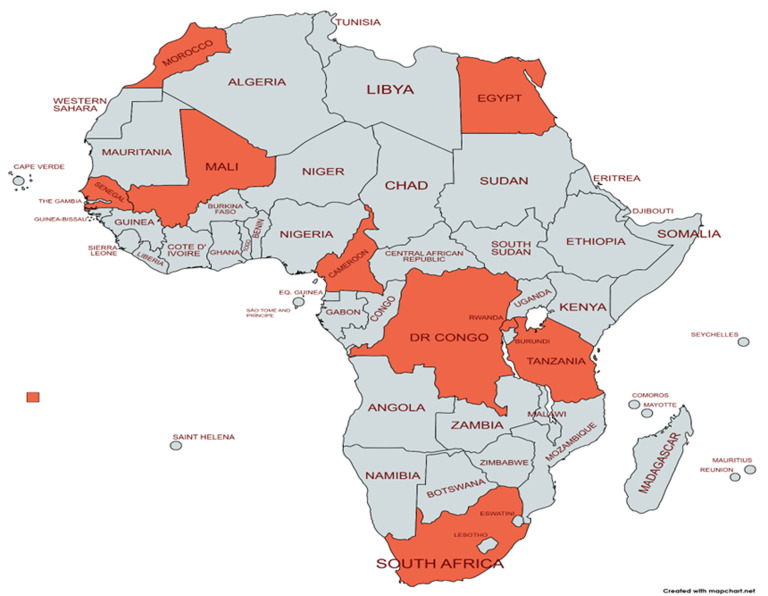
African countries with national human genetics societies affiliated with the African Society of Human Genetics

The African Society of Human Genetics (AfSHG) has successfully integrated its annual meetings and conferences with those of existing national genetics societies as an important catalyst in the formation of new national human genetics societies in Africa since 2003. National human genetics societies affiliated with the AfSHG (Figure 1) have been strengthened through hosting these AfSHG meetings, including the Egyptian, Cameroonian, Democratic Republic of Congo, Senegalese, Malian, Moroccan, and recently, the Tanzanian Society of Human Genetics [1-9].

The African Society of Human Genetics has also been a catalyst for the formation of initiatives that have been significant to the advancement of human genetics in the African continent. For example, the Human Heredity and Health in Africa Consortium (H3Africa) [10,11], Pan African Bioinformatics Network for Africa (H3ABioNet) [12] and most recently, the Data Science for Health Discovery and Innovation in Africa (DS-I Africa) Initiative [13] owe their formation at least in part to the networking platform of scientists provided by AfSHG. These initiatives demonstrate the potential of Africa's contribution to realizing the benefits of genomics in improving healthcare not only in the continent but worldwide. Along these lines, the discussion during the 13^th^ AfSHG conference, which was hosted alongside the 1^st^ conference of the Tanzania Society of Human Genetics (TSHG) now known as Tanzania Human Genetics Organization (THGO), focused on how best to harness the existing, ongoing and future human genetics initiatives to improve health in Africa.

## Thirteenth African Society of Human Genetics (AfSHG) conference

The conference was originally scheduled for 2020 but was postponed to August 2021 due to the COVID-19 pandemic. The meeting was subsequently hosted as a hybrid event with most participants attending online and a small team attending in-person in Dar es Salaam, Tanzania. The meeting was conducted in conjunction with the 18^th^ H3Africa meeting. The main goal of the conference was to discuss the engagement of modern genomic technologies in an African context, particularly the translation to a clinical setting. Talks and sessions were structured around specific themes, including genomic medicine, pharmacogenomics, bioinformatics, and genetic diagnostics for both common and rare genetic disorders (Annex 1).

Over the past ten years, there have been numerous discussions and opinions about how Africa could benefit from the advances in genomics. One school of thought stipulates the need for Africa to focus on basic/primary health interventions, while others think it is necessary for Africa to also participate in the utilization of genomic advancements. Although healthcare priorities are many and significant hurdles exist in incorporating genomics into clinical care in Africa, health genomics in practice continues to rise globally, especially as it becomes more cost-effective. The sharp decrease in the cost of next-generation sequencing now allows researchers in Africa to take part in the human genomics revolution. Given the immense genetic diversity harbored in the African population, the impact of human genetics research in the continent is expected to human genetics research there is expected to contribute to the global healthcare genomics by uncovering new variants with potential health impacts unique to this population [14-16].

**Figure 2:**
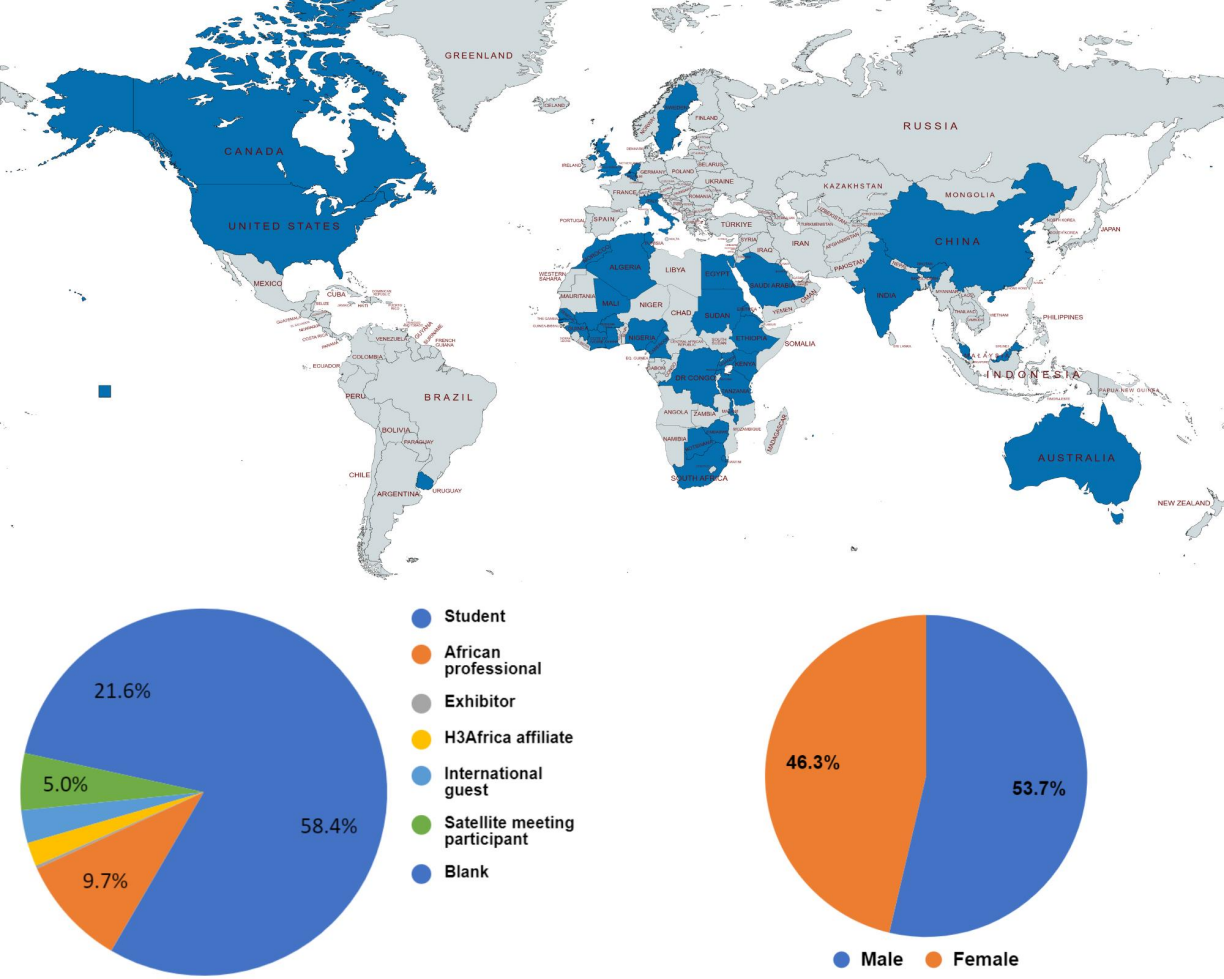
a global map showing the countries of residence of people who registered for the meeting

The conference attracted 380 virtual participants from 40 countries worldwide (Figure 2) and 40 in-person participants, making it one of the largest AfSHG meetings to date. Talks were pre-recorded and made available online to mitigate possible technology and internet challenges that commonly interrupt virtual meetings and to ensure flexibility for participants from different time zones. This was also done for poster presentations, which were made available online throughout the event. To allow an effective engagement, speakers, and presenters were online during their scheduled times for poster presentations. The talks are archived on YouTube.

## The conference program

This conference featured a high engagement of early career researchers (ECRs) in all aspects of the conference from preparation, poster sessions, the pre-meeting workshop, sessions chairing, and presentations. Similar to other AfSHG meetings, this conference was also preceded by a young investigators´ forum (YIF), a platform designed to engage early career researchers by building skills and allowing them to share their work. Opening the workshop, the president of TSHG, Dr. Siana Nkya, reiterated the importance of training in building human capacity for advancing human genomics in the continent. During the forum, participants participated in a short course on human and mammalian genetics, facilitated by Dr. Charlie Wray who leads the education component of the Jackson Laboratory. The course covered three main topics; an up-to-date presentation of genetics in experimental animals and humans, the relationship of heredity to disease in experimental animals and humans, and the importance of molecular genetics in the diagnosis and treatment of inherited disorders. The young investigator´s forum also included an Ensembl genome browser workshop whose primary aim was familiarization with the Ensembl REST API for language-agnostic programmatic access to Ensembl data. This part of the workshop was facilitated by Dr. Vicky Nembaware, the secretary of AfSHG, and Dr. Samson Kilaza, the vice president of TSHG.

The opening session of the conference consisted of welcoming remarks from presidents of the hosting organizations, Dr. Siana Nkya from TSHG and Prof. Ambroise Wonkam from AfSHG, as well as the chairperson of H3Africa, Prof. Christian Happi. During the opening remarks from TSHG, Dr. Nkya emphasized on the importance of platforms such as the conference in bringing together stakeholders to push the human genetics agenda forward. TSHG also highlighted the need of Africa as a continent to realize the promise of genomics translation in improving the health of its people as in other parts of the world. The remarks expressed hope that the conference would be an avenue for discussing how the growing interest and activities in research and application of modern genomic technologies in an African context will lead to translation into clinical practice in the near future.

The president of AfSHG highlighted one of the main achievements of the society i.e. effective capacity building in human genetics in Africa since 2003. AfSHG has been a strong vehicle for networking and connecting African researchers with each other and linking Africa with the rest of the globe to enhance human genetics through research and beyond. On the other hand, the message from the H3Africa consortium chairperson highlighted the great work being done by the consortium and the sustainability it brings through many current and future projects that have and continue to generate accessible datasets for many purposes including future research, opportunities for training, and visibility. He reiterated how the recent studies uncovering new genetic variants have highlighted the diversity and history of the African continent and its people. Further impact of the consortium in mobilizing the first responsive team during the COVID-19 pandemic by using new genetic variants' data and information to plan and execute a safe public response was a significant milestone shared.

The government of Tanzania through the guest of honour, Hon. Mr. Nassor Ahmed Mazurui, the minister of health, social welfare, elders, gender, and children in Zanzibar, acknowledged the importance of human genetics in addressing both communicable and non-communicable health challenges in Tanzania and Africa at large. Hon. Mazrui reaffirmed the government´s support for activities geared towards research and application of human genetics in the country by strengthening local capacity for research and development of research towards discovery, optimization, and deployment of genetic health interventions. Hon. Dr. Jakaya Mrisho Kikwete, the former president of the United Republic of Tanzania and champion of human genetics, was a special guest at the conference. He was represented by the now Ambassador Togolani Mavura. In his message, he acknowledged the role of genetics in defining disease prevalence, distribution, vulnerability, progression, and treatment outcomes of communicable and non-communicable diseases that affect social and economic development. He emphasized the importance of harnessing the understanding of genetics´ role in improving health, underscoring that building capacity in human genetics in the continent affects the entire world, not only Africa.

## Research highlights

The conference featured 6 plenary sessions, covering topics ranging from genetics of host-pathogen coevolution, data science, and genomics in Africa as well as genomics education in Africa. Broadly, the topics were focused on: i) Genomics of infectious diseases including immunodeficiency virus (HIV), Ebola, and coronavirus disease (COVID-19), and; ii) genomics of non-communicable diseases including cardiovascular diseases and hemoglobinopathies including sickle cell disease. Eight (8) parallel sessions were held that gave an opportunity for African researchers to share their research activities and findings on various topics, including: i) Population genetics that highlighted research on genetic variation and associated diseases in African populations; ii) translational research and pharmacogenomics that included topics on translational research for childhood complex disease, genetics of infectious diseases and highlighted challenges on genomics risk prediction focusing on the African polygenic risk scores; iii) developmental disorders in Africa that highlighted research on evaluating clinical exome sequencing in an African setting, genetic diagnosis, genetics of rare diseases such as spinal muscular atrophy as well as congenital abnormalities; iv) cancer genetics and genomics session covered research in hereditary or familial colorectal cancer and genetics of breast cancer; v) genetics of rare diseases that included topics on genodermatosis, perching syndrome, and muscular dystrophy; vi) community engagement and Ethical, Legal, and Social Implications (ELSI) that for the brought on board patients advocacy groups to share their perspectives and research priorities; vii) genetics of infectious disease that focused on the genomics of Malaria, HIV, and tuberculosis, and; viii) genetics and genomics of non-communicable diseases which highlighted research in Mendelian diseases and genetic basis of obesity.

A highlight of this year´s conference was the involvement of patients' voices through patient advocacy groups. Patient advocacy groups representing sickle cell diseases (sickle cell warriors), muscular dystrophy (Ali Kimara Rare Diseases Foundation (AKRDF)), and lupus (Lupus Warriors Tanzania) emphasized the importance of community engagement in human genetics research. Patients indicated the need for concerted efforts to research the incidence and prevalence of rare diseases in Africa apart from research on diagnosis and management of the conditions. Such data is currently missing, hampering the prioritization of policy measures and policy interventions to combat rare diseases. A talk on trauma-related and neutral false memories and associated biological markers in patients with post-traumatic stress disorders following exposure to the 1994 genocide against the Tutsi was also featured in the session. This session was a shift from the scientific talks and attracted attention from the participants, emphasizing how current research can impact population health.

Further, a special issue of the Lancet Haematology series was launched under the theme “priorities in haematological care in sub-Saharan Africa'' during the conference. The goal of this special series was to highlight research on iron deficiency anaemia, sickle cell disease, and haematological cancers, all common conditions with high morbidity and mortality in Africa. These conditions were also selected because they impose a substantial burden on already resource-constrained healthcare systems across the continent [17,18]. The launch included an overview from the guest editor, Prof. Julie Makani, followed by specific talks highlighting updates on iron-deficiency anaemia, blood cancer, sickle cell disease, and blood transfusion studies.

## Meeting operational highlights

**Virtual participation logistics:** the 13^th^ AfSHG conference introduced a distinctive element, the first-ever virtual participation mainly due to travel restrictions because of the COVID-19 pandemic at that time. As a result, this allowed a larger number of participants from diverse geographical locations and professional backgrounds than before (Figure 1). The experience of a virtual platform was enhanced through pre-recorded talks and electronic posters. The pre-recording of talks also proved to be a solution for internet connectivity issues particularly those affecting the speakers. Scientific posters were exhibited digitally for the entire duration of the event, allowing constant access by participants. A speed poster competition during the YIF was an exception because it was live. It also gave early career researchers an opportunity to showcase their work and win prizes.

Experience from this meeting shows virtual conference platforms can be effectively used for scientific conferences in Africa despite the low internet bandwidth there [19]. Technology advances allow effective participation and interaction digitally, either in hybrid mode or fully virtual. Pre-recording talks and posters ensured that the program flowed smoothly and provided increased flexibility for participants across time zones or those who could not travel to participate in person for various reasons. Virtual and hybrid meetings are advocated to enhance scholarly communication and exchange that is more open, inclusive, and less restrictive to the ability to travel [20]. In addition, utilizing a local hub model like that used by H3ABioNet courses may be relevant for AfSHG because member societies already exist in many African countries. This could facilitate other diverse aspects of meetings, especially networking. As the cost of digital meetings continues to fall, we expect the participants´ experience to be enhanced and to accommodate people with special needs.

An important objective of the meeting was to foster public engagement. Public awareness and support is critical in ensuring the success of human genetics initiatives, ensuring a participatory approach, buy in as well as allowing the public to make informed choices with genetic products and services that result from human genetics research. Media engagement prior to, during, and post this meeting showed us that a carefully curated blend of mass and social media engagement is key to reaching a diverse audience. We also learned that crafting key messages in advance and repeatedly communicating those in a language fit for different platforms depending on specific audiences was also important in spreading the message. Media engagement amplified the message of the conference and left a positive impression on the public as evidenced by ensuing discussion on different platforms and feedback from people who were reached.

## Way forward and future perspectives

African Society of Human Genetics meetings play a key role in highlighting ongoing human genetics initiatives that inform research and healthcare priorities, resources, and gaps across the continent. To date, AfSHG meetings have been a great catalyst to advance human genetics activities in Africa. Consecutive AfSHG meetings are planned to continue showcasing human genetics activities in Africa as well as building capacity across the continent. Following the conclusion of the 13^th^ AfSHG conference, the 14^th^ conference with the theme “applications of genomics medicine in Africa” was scheduled to take place in conjunction with the 2^nd^ International Congress of the Moroccan Society of Genomics and Human Genetics (SM2GH) on 12^th^ to the 17^th^ December, 2022, at the Mohammed VI Foundation Conference center, Rabat, Morocco.

**Figure 3:**
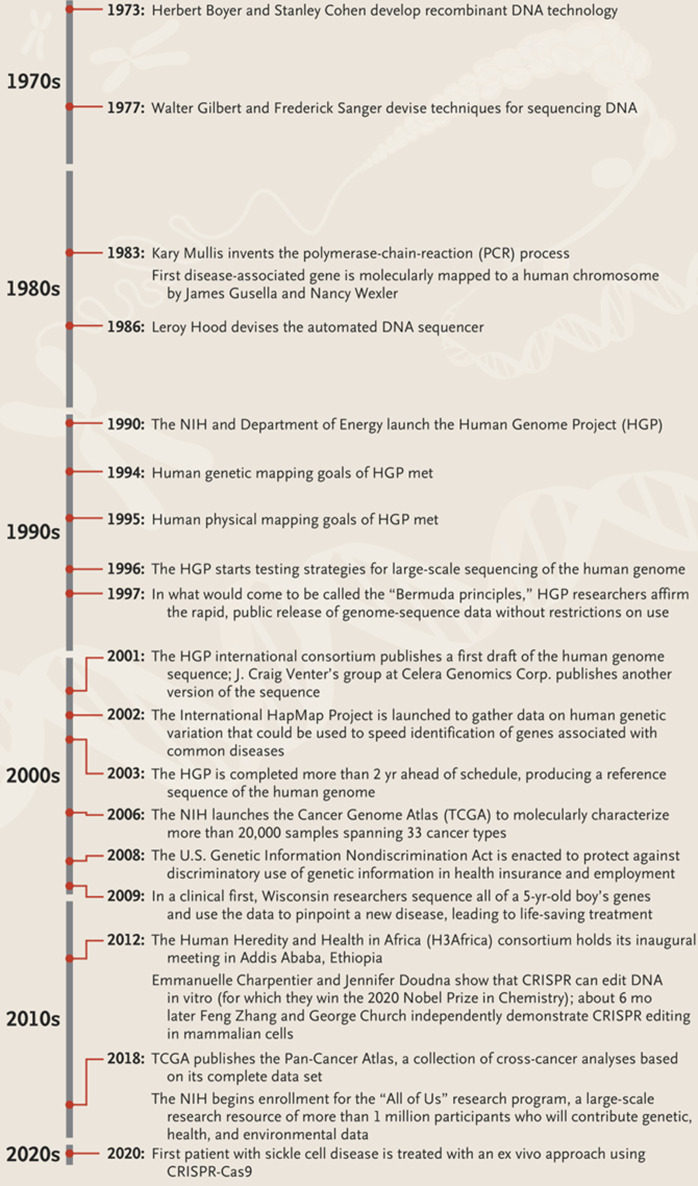
highlights of important milestones in the global history of human genetics (Collins *et al*. 2021)

In their recent article titled “human molecular genetics and genomics-important advances and exciting possibilities”, [21] highlighted important human genetics milestones to date (Figure 3). In these milestones, the biggest question that remains unanswered is how Africa can be better positioned to equally harness these advancements in human genetics to improve the health of her people like the rest of the world. Larger cohorts for genetic studies and research at different levels from basic, translational, and applied to implementation studies will be required to address this gap. It is thus necessary to ensure that Africa is participating in all stages of the human genetics global agenda including the planning and execution. We hope that this meeting contributed to the discussion on how Africa can harness the promise of human genetics hidden in her wealth of genetic variation and diversity, not only for the wellbeing of her people but also for the entire world.

## Annex

**Annex 1**: the program for the 13^th^ African Society of Human Genetics conference (PDF-114Kb)
